# Neural Complexity of Implicit Attitudes Predicts Exercise Behavior in Hypertensive Patients: An EEG Entropy Study

**DOI:** 10.3390/brainsci16020244

**Published:** 2026-02-22

**Authors:** Xingyi Tang, Chengzhen Wu, Haoming Ma, Bo Yao, Ting Li, Meihua Piao

**Affiliations:** 1 School of Nursing, Chinese Academy of Medical Sciences & Peking Union Medical College, Beijing 100144, China; tangxingyi@pumc.edu.cn (X.T.); haoming_ma@student.pumc.edu.cn (H.M.); 2 Center for Cognitive and Brain Sciences, Institute of Collaborative Innovation, University of Macau, Macau 999078, China; yc57945@um.edu.mo; 3 Institute of Biomedical Engineering, Chinese Academy of Medical Sciences & Peking Union Medical College, Tianjin 300192, China; aragon_y@outlook.com (B.Y.); liting@bme.cams.cn (T.L.)

**Keywords:** implicit attitude, exercise, entropy, EEG

## Abstract

**Highlights:**

**What are the main findings?**

- EEG entropy during implicit attitude processing showed stronger discrimination of subsequent exercise behavior than traditional reaction time-based D-scores in patients with hypertension.
- Among different task conditions, envelope entropy features derived from affective incompatible IAT conditions demonstrated the most consistent differences between exercisers and non-exercisers.

**What are the implications of the main findings?**

- Neural complexity metrics provide a complementary and interpretable perspective for understanding implicit attitude processing underlying exercise behavior beyond behavioral reaction time measures.
- These findings highlight the potential value of incorporating neural complexity markers of implicit processing into future models of exercise behavior, while underscoring the need for validation in larger and more diverse samples.

**Abstract:**

Background: Exercise is a key component in managing hypertension, yet adherence remains low. Beyond deliberate decision-making, implicit attitudes also play an important role in exercise behavior as automatic and unconscious evaluative processes. Traditional studies mostly rely on reaction time measures, which are susceptible to practice effects and fail to capture dynamic neural processing. Objectives: This study aimed to examine whether the EEG entropy derived from implicit attitude processing can better predict exercise behavior than traditional reaction time measures in patients with hypertension. Methods: Fifty-seven hypertensive patients completed affective and instrumental implicit association tests (IATs) with EEG recording. Seven entropy features were extracted. Multiple machine learning algorithms were applied to compare the predictive performance of reaction time with EEG entropy features. The random forest model was used to analyze the importance ranking of features from different brain regions. Results: EEG entropy outperformed reaction times in distinguishing exercisers from non-exercisers. Affective implicit attitudes consistently demonstrated stronger accuracy than instrumental attitudes. Envelope entropy showed the most robust and significant group differences. For the random forest (RF) classifier of envelope entropy, classification accuracies were 71.9% for the affective IAT (incompatible task only), and 71.9% for the model combining affective and instrumental IAT features. Frontal and central regions contributed most to classification. Conclusions: EEG entropy, particularly envelope entropy during affective IAT-incompatible tasks, provides superior discrimination of exercise behavior than reaction time measures. This suggests that exercise behavior is closely linked to the neural complexity underlying affective conflict processing. These findings advance our understanding of the neural dynamic patterns linking implicit attitudes and exercise behavior and suggest EEG entropy as a promising tool for assessing and intervening exercise behavior.

## 1. Introduction

Moderate-to-vigorous exercise is an important non-pharmacological strategy for hypertension management, with well-established benefits for cardiovascular function, metabolic health, and the reduction in cardiovascular disease risk [1]. Nevertheless, a substantial proportion of patients with hypertension fail to engage in exercise in daily life [2]. Researchers have attempted to explain exercise behavior through constructs such as perceived benefits, attitudes, intentions, and self-efficacy. However, the explanatory power of these variables remains limited, particularly in populations with chronic diseases [3,4]. Intention–behavior gaps still exist, that individuals report strong intentions to exercise but do not translate these intentions into actual behavior [5]. Most of these explanatory variables originate from rational health behavior models, including the Theory of Planned Behavior and the Health Belief Model, and are primarily assessed through self-report measures following deliberate evaluation [6]. In contrast, dual-process theory posits that human behavior is jointly shaped by explicit (deliberate, conscious) and implicit (automatic, unconscious) processes [7]. To better account for exercise behavior beyond reflective decision-making, recent research has increasingly focused on automatic and implicit processes and their behavioral relevance.

Implicit attitudes are thought as automatic evaluative responses shaped by long-term socialization experiences and affective association, capable of influencing behavioral tendencies in the absence of conscious control [8,9]. They typically comprise affective and instrumental components. Affective implicit attitudes reflect automatic associations between a target object and affection (e.g., pleasant/unpleasant), whereas instrumental implicit attitudes capture associations with functional or value-based concepts (e.g., useful/useless, important/unimportant) [10,11,12]. The Implicit Association Test (IAT) assesses implicit attitudes by measuring reaction time differences between compatible and incompatible conditions and is considered less susceptible to social desirability and self-report biases than explicit questionnaires [13]. Previous studies have reported associations between affective implicit attitudes [14,15], instrumental implicit attitudes [10,16], and exercise behavior or differences between individuals with varying exercise levels. However, there are also studies that have not found significant results [17,18]. It has also been found that affective implicit attitude can significantly predict exercise, while instrumental implicit attitudes cannot [10,11]. Moreover, most existing studies have focused on healthy young adults, with limited attention to clinical populations such as patients with hypertension. Recent meta-analysis indicates a small but significant association between implicit attitudes and physical activity [19]. Notably, reaction time-based measures may be influenced by practice effects and strategic responding, potentially compromising measurement validity [20]. As an endpoint behavioral output, reaction time also provides limited insight into the underlying automatic processing dynamics.

To gain a more comprehensive understanding of the relationship between implicit attitudes and exercise behavior, several studies have combined IAT paradigms with electroencephalography (EEG), primarily using event-related potentials (ERPs) to examine temporal components of neural processing. For example, one study found that regular runners exhibited larger N400 differences between compatible and incompatible affective IAT conditions than irregular or non-runners, suggesting reduced semantic conflict for positive associations, while no group differences were observed in the late positive potential (LPP) [21]. Another study reported significant group differences in the P2 component during instrumental IAT tasks, whereas N1 and P3 components did not differentiate individuals with different exercise levels [10]. Although ERP approaches provide valuable information about temporally specific neural components, they inherently focus on linear, phase-locked activity and emphasize discrete processing stages. However, implicit attitudes are fast, automatic evaluative processes that may emerge from dynamic interaction and competition of multiple neural processes [22]. Such distributed and fluctuating neural dynamics may be more relevant for understanding future exercise behavior than the amplitude of single ERP components at specific latencies. Therefore, it is necessary to investigate how the overall stability, flexibility, and organization of neural processing states influence exercise behavior.

Entropy-based metrics offer a complementary perspective for characterizing such neural processing by quantifying nonlinear dynamics and signal complexity, reflecting the predictability and variability of neural activity during information processing [23]. Unlike traditional ERP or frequency domain analyses, entropy emphasizes system-level dynamics and provides a global description of neural processing organization. Higher entropy is generally interpreted as indicating greater neural complexity, flexibility and adaptivity, whereas lower entropy reflects more constrained or stereotyped processing patterns [24,25]. EEG can be seen as neural responses to events or stimuli in IAT [26]. Implicit attitudes processing may rapidly integrate many components and stages, such as attention and conflict monitoring [22]. Accordingly, EEG entropy may be interpreted as a marker of neural dynamic complexity and uncertainty during implicit attitude processing, providing insights of how automatic evaluations are neurally organized rather than their strength. Affective and instrumental implicit attitudes may differ in their development, neural constructs and bases [27,28]. Affective implicit attitudes are more strongly grounded in affect-related neural components, including emotional appraisal and motivational systems, whereas instrumental implicit attitudes are more closely linked to cognitive components involved in semantic processing, value evaluation, and goal-directed control. Neural entropy may therefore capture different organizational patterns during affective and instrumental implicit attitude processing. Consequently, the relationship between entropy-based neural dynamics and subsequent exercise behavior may also differ between these two forms of implicit attitudes.

Entropy measures have been widely applied to EEG data in both resting-state and task-based paradigms. They have been used to differentiate neural complexity in bipolar disorder [29], Alzheimer’s disease [30], and Parkinson’s disease [31], as well as to classify emotion [32]. It has also been applied to cognitive tasks such as the psychomotor vigilance tests [33] and sustained attention tasks [34], and to motor imagery paradigms to capture internal motor-related processing without actual movement [35,36]. Moreover, higher levels of physical activity have been associated with increased multiscale entropy during visuo-spatial cognition tasks, suggesting a link between exercise behavior and neural information processing dynamics [37]. Despite these advances, entropy has not yet been systematically integrated into IATs to investigate the neural complexity of implicit attitude processing and its relationship with actual exercise behavior. It is common to encounter unhappy or conflicting situations during exercise, and it is crucial to carry out self-regulation [18]. Studies have shown that higher entropy is associated with stronger self-regulation [25]. Therefore, analyzing exercise behavior using the entropy characteristics of implicit attitude processing may provide new perspectives.

Machine learning approaches have increasingly been applied to neural data to model complex, high-dimensional patterns and to predict cognitive, affective or behavioral measures from distributed brain activity [38]. A growing body of research has demonstrated that such approaches can effectively decode information from neural signals [39,40,41]. However, the potential value of neural dynamic features for decoding exercise behaviors, particularly those grounded in implicit evaluative processing, remains relatively limited. Therefore, integrating EEG entropy features with machine learning classification provides a data-driven approach to examine whether neural dynamics during implicit attitude processing can discriminate subsequent exercise behavior.

Based on these theoretical and empirical considerations, this study aimed to explore whether the EEG entropy characteristics derived from affective and instrumental IATs can better predict exercise behavior than the traditional D-score in patients with hypertension. Specifically, we hypothesized that: (1) entropy-based neural complexity features would yield higher classification accuracy for exercise behavior than traditional reaction time-based D scores; (2) entropy features derived from affective IATs would demonstrate stronger discriminative power for exercise behavior than those derived from instrumental IATs; (3) entropy measured during incompatible conditions would be more discriminative of exercise behavior than entropy measured during compatible conditions; and (4) entropy features from central region that related to motor would contribute more strongly to exercise classification than other regions. By adopting a neural dynamical perspective, this study complements traditional behavioral and linear neural indices and advances understanding of the neurodynamic correlates of exercise behavior during implicit attitude processing.

## 2. Materials and Methods

### 2.1. Participants

From June to September 2025, 57 hypertensive patients (mean age = 46.16 years, SD = 9.77) were recruited from community health centers. Their characteristics are summarized in Table 1. Inclusion criteria were: age 18–60 years, diagnosed with primary hypertension, normal finger function, no caffeine intake within the previous 12 h, at least 8 h of sleep the night before testing, and no antihypertensive medication intake within the previous 2 h. Exclusion criteria included: cognitive, psychiatric, or sensory impairments; neurological disorders; inability to perform exercise; or participation in other studies within the past three months. These conditions were confirmed through self-report at the beginning of the experiment. Written informed consent was obtained from all participants, and the study was approved by the Institutional Review Board (PUMCSON-2025-20; approval date: 30 June 2025).

### 2.2. Measurements

Affective and instrumental implicit attitudes toward exercise were assessed using Single Category Implicit Association Tests (IATs). Following previous studies [10,11,12], we selected target and attribute words, and 30 hypertensive patients rated the valence (1 = perfectly negative, 9 = perfectly positive) and relevance of these words to “exercise” and “positive/negative affective/instrumental aspects of exercise” (1 = not at all descriptive, 9 = perfectly descriptive) on a 9-point scale. The six highest-rated words in each category were selected as the final stimuli (Appendix A). IATs were programmed in E-Prime 2.0 and included compatible and incompatible blocks, with 24 practice trials and 72 formal trials per block. Participants were instructed to categorize stimuli by pressing keys according to the instructions (see Table 2). Each trial began with a fixation cross (“+”, 500 ms), followed by the stimulus word (1500 ms) during which participants responded as quickly and accurately as possible. Correct responses in practice trials were indicated by “√” (150 ms) and incorrect or missed responses by “×”. A blank screen (1000 ms) separated trials. Feedback was omitted in formal trials.

Seven-day recall of exercise was assessed using the “recreation, sport, and leisure-time physical activity” section of the International Physical Activity Questionnaire (IPAQ) [42]. Participants reported the number of days per week and the average daily duration of walking, moderate-intensity, and vigorous-intensity exercises performed during the previous seven days. Weekly energy expenditure was calculated in MET-min/week by multiplying the MET (Metabolic Equivalent of Task) value assigned to each exercise (walking = 3.3 METs; moderate = 4 METs; vigorous = 8 METs) by the corresponding minutes per day and days per week.

EEG data were recorded using 64-channel AgCl scalp electrodes placed according to the standard 10–20 system. Signals were amplified via a portable wireless EEG system (NeuSen.W64, Neuracle, Changzhou, China) at a sampling rate of 1000 Hz. All electrode impedances were kept below 10 kΩ.

### 2.3. Procedure

Participants completed affective and instrumental IATs in a quiet environment while EEG data were simultaneously recorded. To control for order effects, the sequence of IAT types and compatible/incompatible tasks was counterbalanced across participants. Each IAT lasted approximately 15 min, with at least a 10-min break between tests. Seven days after the experiment, participants completed the IPAQ online to report their exercise.

### 2.4. Data Processing and Feature Extraction

#### 2.4.1. D-Score Calculation from IAT Reaction Times

Practice trials were excluded. Participants with error rates exceeding 20% for any task were removed. Trials with reaction times <350 ms or missing responses were discarded. For incorrect trials, reaction time was replaced by the mean of correct trials in the same block plus 400 ms. D-scores were calculated by subtracting mean compatible trial reaction times from incompatible trial reaction times, divided by the pooled standard deviation of correct trials [13].

#### 2.4.2. EEG Preprocessing

Only formal trials with correct responses were analyzed. EEG preprocessing was conducted in MATLAB R2018b using EEGLAB toolkit [43]. The data were re-referenced offline to the average of the bilateral mastoids. Based on previous studies [29,44], the continuous EEG data were downsampled to 500 Hz and band-pass filtered between 3 and 40 Hz using a finite impulse response (FIR) filter. Epochs were extracted from −500 to 1000 ms relative to stimulus onset, with a baseline correction of −200 to 0 ms. Bad channels and epochs were identified based on abnormal signal characteristics, including excessively high variance, persistent high-amplitude noise, or flat-line signals, as determined by visual inspection. Bad channels were interpolated using spherical spline interpolation when they were outside the regions of interest (details in Section 2.4.3). If a bad channel belonged to the regions of interest, the data was excluded from further analysis. To isolate and remove ocular and muscular artifacts, Independent Component Analysis (ICA) was performed using the logistic Infomax ICA algorithm. Independent components (ICs) representing artifacts were identified based on their scalp topographies, time courses, and power spectral characteristics. Components associated with eye blinks and muscle noise were removed after visual inspection by two experienced researchers. Finally, epochs with amplitudes exceeding ±100 μV were rejected.

#### 2.4.3. Entropy Feature Extraction

We selected seven entropy metrics applied in motor-related and EEG research: singular spectrum entropy, approximate entropy, sample entropy, fuzzy entropy, permutation entropy, envelope entropy, and log energy entropy [44]. The specific formulas and meanings of entropy are given in Appendix B. The window width of singular value decomposition for singular spectrum entropy was set to 500. For approximate entropy, sample entropy and fuzzy entropy, the embedding dimension was 2 and the threshold was 0.15. For permutation entropy, the embedding dimension was 6 and the time delay was 1 [44]. Features were computed for four IAT conditions (affective compatible/incompatible, instrumental compatible/incompatible) across five brain regions: frontal (Fz, F1, F2), fronto-central (FCz, FC1, FC2), central (Cz, C1, C2), centro-parietal (CP1, CP2), and parietal (Pz, P3, P4) regions. Regional entropy values were obtained by averaging across representative electrodes to mitigate random variability at the single-channel level and to enhance the reliability of entropy-based neural features.

### 2.5. Data Analysis and Classification

In the present study, exercise behavior was operationalized as the presence versus absence of moderate-to-vigorous exercise during the 7-day follow-up period, given the importance of initiating moderate-to-vigorous exercise and its established relevance for cardiovascular health in hypertension [1]. Walking was therefore not included. Participants were classified as the non-exercise group if their total moderate-to-vigorous exercise equaled 0 MET-min/week during the 7-day follow-up, and as the exercise group if their total moderate-to-vigorous exercise exceeded 0 MET-min/week. To evaluate the discriminative value of implicit attitudes at the behavioral level, random forest classifiers were first constructed using IAT D-scores as predictive features. In parallel, entropy-based classification models were established using seven entropy features extracted from five brain regions. Separate models were developed based on affective IAT, instrumental IAT, and their combination. This high-dimensional analysis served as an exploratory baseline to compare the relative information content of neural complexity measures versus D-scores, rather than as a final optimized predictive model. The results should be interpreted cautiously due to the potential risk of overfitting.

To facilitate interpretation among complexity indices, a subsequent metric-focused dimensionality reduction was performed. Specifically, Wilcoxon rank-sum tests were conducted to compare entropy values between the exercise and non-exercise groups. They were applied to describe group-wise differences in entropy patterns across task conditions and brain regions. To account for multiple comparisons, false discovery rate (FDR) correction was applied using the Benjamini–Hochberg procedure. For each IAT task condition, all entropy–region combinations (5 regions × 7 entropy metrics = 35 tests) were treated as a single family. The control level set at q = 0.05. This task-wise FDR control was chosen because different IAT conditions reflect theoretically distinct cognitive–affective processing contexts and should not be pooled into a single family. These analyses were used to compare the relative stability and behavioral relevance of different entropy metrics, rather than to perform strict supervised feature selection.

Based on the entropy metric identified through this comparative evaluation, multiple classification models were constructed using different combinations of IAT type and task condition, including: compatible and incompatible tasks of affective IAT, compatible tasks of affective IAT, incompatible tasks of affective IAT, compatible and incompatible tasks of instrumental IAT, compatible tasks of instrumental IAT, incompatible tasks of instrumental IAT, and the combined affective and instrumental IAT (compatible and incompatible tasks combined). For each feature set, four machine learning algorithms—random forest (RF), k-nearest neighbors (KNN), support vector machine (SVM), and linear discriminant analysis (LDA)—were applied to classify exercise behavior. Feature importance was further examined using the random forest models. The five most informative features were identified based on their importance scores. To facilitate comparison across features, importance values were normalized by calculating the proportional contribution of each feature relative to the total importance of the top five features. For model implementation, the random forest classifier consisted of 100 trees, with the minimum number of samples per leaf set to 5 to mitigate overfitting. The KNN classifier used 5 nearest neighbors with Euclidean distance as the distance metric. The SVM classifier employed a radial basis function (RBF) kernel with an automatically optimized kernel scale and a box constraint parameter set to 1. Data were randomly split into training (70%) and testing (30%) sets. To reduce variability due to random data partitioning, all classification procedures were evaluated using ten-fold cross-validation. Model performance was evaluated using confusion matrices, from which accuracy, sensitivity, specificity, precision, and F1-score were calculated. For each model, all performance metrics were reported as the mean ± standard deviation across the 10-fold cross-validation to reflect both discriminative ability and model stability. Receiver operating characteristic (ROC) curves and the area under the curve (AUC) were presented for the primary classification models that support the main conclusions, to avoid redundancy across highly similar classifiers. In all classification analyses, the non-exercise group was defined as the positive class.

## 3. Results

**Figure 1:**
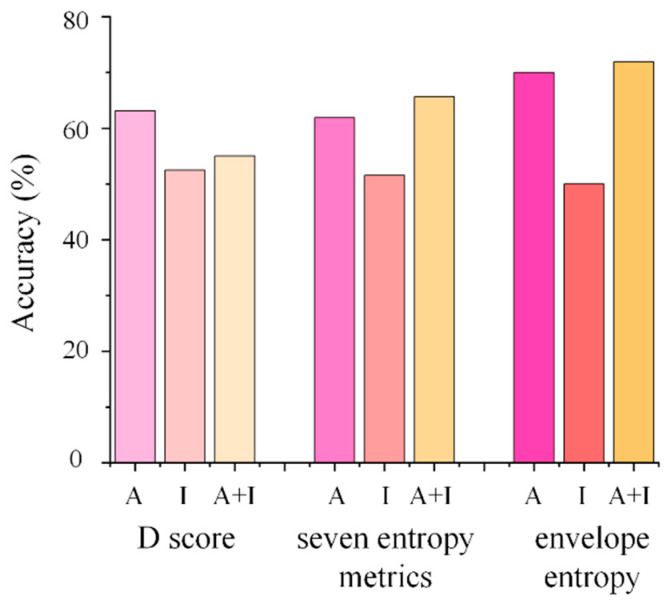
Classification accuracy of D score, seven entropy metrics, and envelope entropy using random forest classifiers. A, affective Implicit Association Test (IAT); I, instrumental IAT; A+I, affective and instrumental IAT.

A total of 57 hypertensive patients were enrolled. One participant was excluded due to poor EEG data quality, resulting in 56 participants included in the final EEG analyses. Of these, 33 participants were classified into the exercise group and 23 into the non-exercise group. Using random forest classifiers based on D-scores in Implicit Association Test (IAT), prediction accuracy for exercise behavior reached 63.1% for the affective IAT, 52.5% for the instrumental IAT, and 55.0% for the combined affective–instrumental model. When seven entropy features from five brain regions were used, classification accuracy was 61.9% for affective IAT, 51.3% for instrumental IAT, and 65.6% for the combined model. About envelope, classification accuracy was 70.0% for affective IAT, 50.0% for instrumental IAT, and 71.9% for the combined model (Figure 1).

Group differences in EEG entropy between exercisers and non-exercisers are summarized in Table 3 and Table 4. In the affective IAT-incompatible task, envelope entropy demonstrated robust and consistent group differences across multiple regions, including the frontal (z = −3.472, *p* = 0.012), fronto-central (z = −3.789, *p* < 0.001), and central regions (z = −3.339, *p* = 0.012).

**Figure 2:**
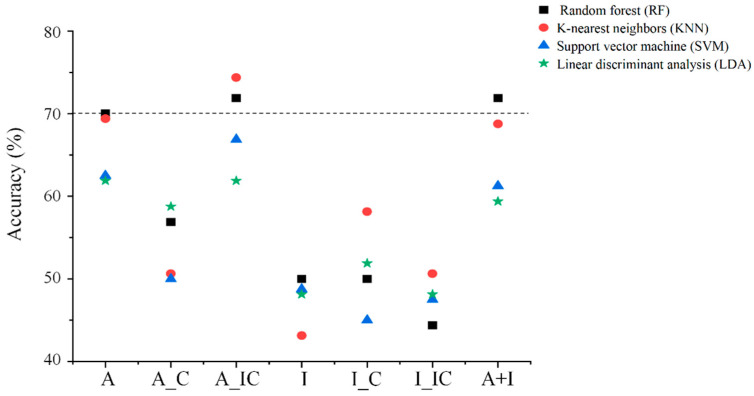
Classification accuracy across different machine learning models using envelope entropy. A, affective IAT; A_C, affective IAT compatible task; A_IC, affective IAT-incompatible task; I, instrumental IAT; I_C, instrumental IAT compatible task; I_IC, instrumental IAT-incompatible task; A+I, affective and instrumental IAT.

**Figure 3:**
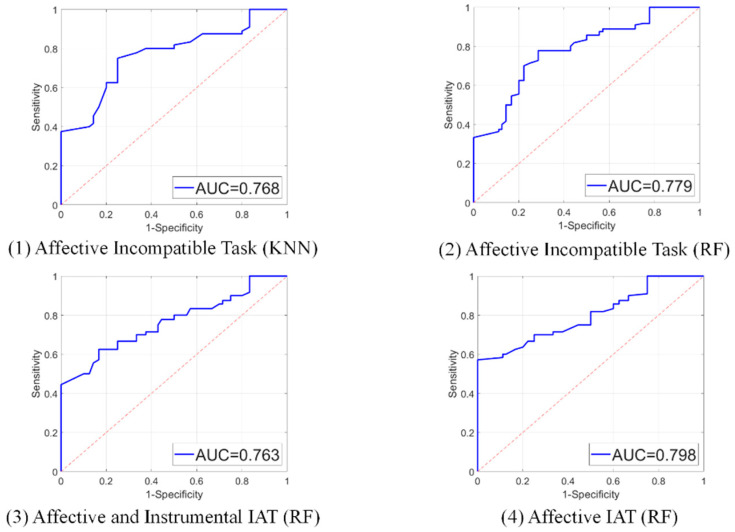
Receiver operating characteristic (ROC) curves and area under the curve (AUC) values for the four best-performing classification models using envelope entropy features. The models correspond to: (**1**) k-Nearest Neighbors for affective IAT-incompatible tasks, (**2**) Random Forest for affective IAT-incompatible task, (**3**) Random Forest for combined affective and instrumental IAT, and (**4**) Random Forest for affective IAT (compatible and incompatible tasks).

**Figure 4:**
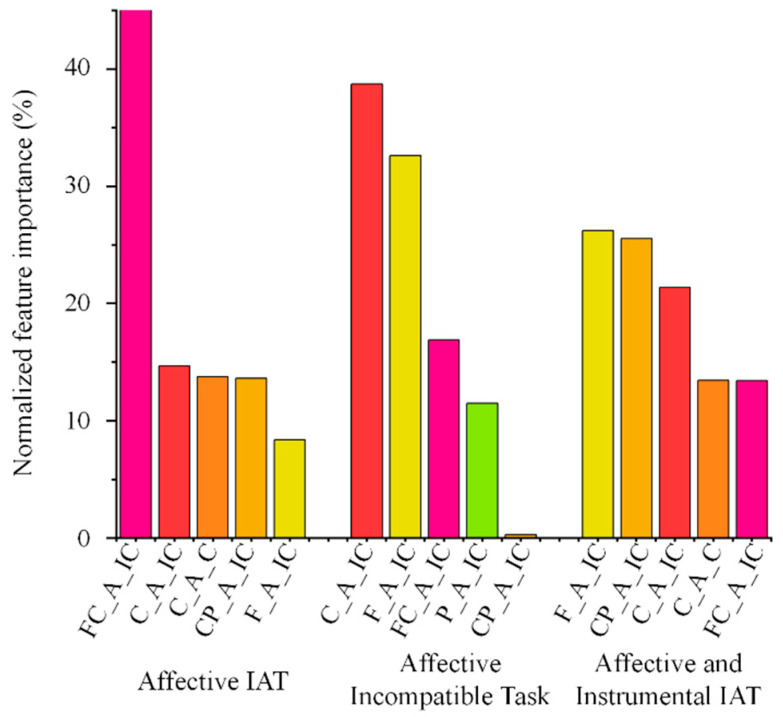
Feature importance ranking (the top five contributing entropy features). FC_A_IC, affective incompatible task in fronto-central region; C_A_IC, affective incompatible task in central region; C_A_C, affective compatible task in central region; CP_A_IC, affective incompatible task in centro-parietal region; F_A_IC, affective incompatible task in frontal region; P_A_IC, affective incompatible task in parietal region.

Based on these findings, envelope entropy features from all five brain regions were used as input variables for subsequent classification analyses across single-task and combined-task conditions. Classification performance across different machine learning models is presented in Table 5 and Figure 2. For the random forest (RF) classifier, classification accuracies were 70.0% for the affective IAT (compatible and incompatible tasks combined), 71.9% for the affective IAT (incompatible task only), and 71.9% for the model combining affective and instrumental IAT features. For the k-nearest neighbors (KNN) classifier, the classification accuracy for the affective IAT (incompatible task only) was 74.4%. The corresponding receiver operating characteristic (ROC) curves and area under the curve (AUC) values are presented in Figure 3. Feature importance rankings from the random forest analyses are shown in Figure 4, highlighting the top five contributing entropy features.

## 4. Discussion

By integrating implicit association tests (IATs), EEG-based entropy measures, and machine learning approaches, this study examined whether neural dynamics during implicit attitude processing can discriminate subsequent exercise behavior in patients with hypertension. The findings indicate that, compared with behavioral reaction-time indices, neural complexity features may provide more sensitive markers for identifying individuals who engage in exercise behavior, particularly under affective conflict conditions. These are largely consistent with our hypothesis.

Across both reaction-time-based and entropy-based models, affective implicit attitudes consistently demonstrated stronger discriminative power than instrumental implicit attitudes. Affective implicit attitudes primarily reflect automatic emotional associations with exercise, whereas instrumental implicit attitudes index automatic cognitive associations between exercise and its functional or outcome-related value [10,11,12]. Much evidence suggests that affective implicit attitudes play a prominent role in shaping exercise behavior [37], whereas findings regarding instrumental implicit attitudes remain limited and inconsistent. It has shown that running students have more positive affective and instrumental implicit attitudes than non-running students [10]. In another study, affective implicit attitude can predict leisure-time physical activity, while instrumental implicit attitudes cannot [11]. These findings collectively suggest that exercise behavior may be predominantly driven through affective pathways, as actual exercise experiences are closely accompanied by immediate pleasurable or painful sensations, and repeated emotional associations are more directly linked to behavioral choice [8].

We found that classification accuracy based on D scores was lower than that achieved using EEG entropy features when predicting moderate-to-vigorous exercise. Although previous research has reported significant associations between implicit attitudes and physical activity, effect sizes are typically small and results remain heterogeneous [19]. Notably, some studies have found no significant differences in D scores between exercisers and non-exercisers, but clear distinctions in neural indicators [21]. It suggests that reaction time may have limited predictive effect, whereas neural measures may offer greater sensitivity. It is possible that the D score represents a terminal behavioral output derived from reaction times and is susceptible to practice effects or response strategies [20]. Moreover, reaction time measures primarily reflect the outcome of implicit processing while providing little information about the underlying evaluative dynamics. Implicit attitudes are likely the result of competing and mutually inhibitory neural processes [22], and neural differences during implicit evaluation may not translate linearly into observable reaction time differences. Therefore, EEG entropy that captures the overall dynamical complexity of neural processing may constitute a more informative approach for investigating the neural dynamic patterns linking implicit attitudes to exercise behavior; this warrants further investigation.

Entropy metrics are widely used to characterize the complexity of EEG signals, while different entropy measures vary in their sensitivity to specific aspects of neural dynamics due to differences in computational principles [35]. Among the multiple entropy indices compared in this study, envelope entropy demonstrated the most stable and robust differences between exercisers and non-exercisers. Unlike entropy measures that primarily rely on temporal structure or pattern recurrence in the raw signal, envelope entropy is derived from the instantaneous amplitude envelope obtained via the Hilbert transform and quantifies the complexity of its distribution [45]. The Hilbert transform enables the construction of an analytic signal from which instantaneous amplitude can be directly extracted, providing a principled means of capturing amplitude modulation without relying on rigid filtering assumptions. When applied to nonlinear and non-stationary EEG signal, envelope-based representations may better reflect neural information embedded in amplitude dynamics rather than in purely temporal regularities [46,47]. This property may be especially relevant for affective implicit evaluation and conflict processing, during which variations in neural activation intensity may reflect how automatic emotional processing translate into behaviors.

Furthermore, classification analyses revealed that envelope entropy derived from affective IAT conditions, particularly the incompatible task, yielded the highest accuracy for subsequent exercise behavior across multiple classifiers. Notably, exercisers exhibited significantly higher envelope entropy than non-exercisers during affective incompatible tasks. EEG entropy is thought to reflect neural flexibility and the capacity to transition between functional brain states [48]. Higher entropy has been associated with more flexible, adaptive, and variable information processing and emotion regulation [25]. From the perspective of dual-process models, these findings suggest that exercise behavior may be closely linked to individual differences in automatic processing, especially the affective conflict regulation. Within this framework, elevated envelope entropy in exercisers may indicate a more flexible neural response when processing negative affective associations (e.g., “exercise–unpleasant”), potentially attenuating the inhibitory influence of automatic negative evaluations on behavior. In contrast, non-exercisers may exhibit more rigid or convergent amplitude dynamics, reflecting less adaptable processing under affective conflict. Importantly, these differences were specific to affective, incompatible task conditions and were not observed in instrumental or compatible tasks, underscoring the task-specific nature of the neurodynamic patterns associated with exercise behavior. This specificity suggests that exercise behavior is more closely linked to neural dynamics during affective conflict processing.

Previous studies have reported high classification accuracies when using EEG entropy features to decode motor imagery-related process, with performance exceeding 90% [49,50]. However, they typically involve neural activity that is closely coupled with immediate motor intentions. In contrast, predicting real-world exercise behavior represents a substantially more complex challenge, as engagement in exercise is shaped by multiple motivational factors. Notably, prior studies predicting physical activity using demographic and lifestyle variables have reported best-performing models with accuracies around 70% [51], which is comparable to our study. Although our accuracies are lower than that achieved in real-time action decoding paradigms, our findings provide evidence that neural dynamic features derived from implicit processing contain behaviorally relevant information in the context of exercise behavior.

Finally, converging evidence from group comparisons and feature importance analyses indicated that frontal and central regions contributed most strongly to exercise classification. The frontal cortex plays a central role in emotion regulation [52], conflict monitoring, and cognitive control [53], whereas central regions are closely associated with motor preparation and action representation [54]. Enhanced envelope entropy within these regions during affective incompatible tasks may reflect more efficient integration of emotional conflict processing and motor readiness in exercisers. Specifically, exercisers may have a more complex implicit emotion network, enabling the frontal region to integrate conflict emotion information more effectively. At the same time, the central region more flexibly links these implicit emotion processing signals with the motor preparation state, thereby presenting a higher envelope entropy in the time-amplitude dynamics.

## 5. Limitations

Several limitations of this study should be considered. First, although a prospective design was adopted, the follow-up period was relatively short and may not fully capture the stability of long-term exercise behavior. Future studies with longer follow-up durations are needed to examine the sustained predictive validity. In addition, exercise behavior was assessed using self-report measures, which are vulnerable to recall and social desirability bias. Future research should incorporate objective measures to improve assessment.

Second, the sample size was modest and consisted exclusively of patients with hypertension, which may limit the generalizability of the findings and increase the risk of overfitting in classification analyses. Accordingly, the results should be interpreted with caution. Moreover, potentially relevant covariates, such as medication type and timing, baseline fitness, and other clinical characteristics, were not included in the current analyses. Future studies with larger samples should statistically control for these factors to more rigorously assess the relationship between implicit neural processing and exercise behavior. And considering alternative exercise classification criteria can better capture variability in exercise behavior.

Finally, the present machine learning analyses were exploratory in nature. The use of multiple entropy features may increase the risk of redundancy and overfitting, and the subsequent focus on envelope entropy was informed by prior statistical comparisons. Although this approach was motivated by interpretability and descriptive considerations, some dependency between feature selection and classification performance cannot be fully excluded. Therefore, the reported classification results should be interpreted as preliminary. Future research with larger samples, fully nested feature selection procedures, and independent validation cohorts is required to further confirm the robustness of these findings.

## 6. Conclusions

This study provides exploratory evidence that EEG entropy during implicit attitude processing is associated with subsequent exercise behavior in hypertensive patients. Compared with reaction-time measures, neural entropy, particularly envelope entropy, demonstrated superior discrimination of exercisers and non-exercisers. Affective implicit attitudes consistently outperformed instrumental implicit attitudes, indicating a closer link between automatic affective evaluations and exercise behavior. The strongest classification performance was observed for envelope entropy during affective IAT-incompatible tasks, with frontal and central regions contributing the most, suggesting that neural dynamics related to affective conflict processing and motor-related representations are critical for distinguishing exercise behavior. Overall, these findings highlight the utility of neural complexity measures in capturing implicit attitudes underlying exercise behavior.

## Figures and Tables

**Table 1:** Characteristics of participants (N = 57).

Variable	Category	Value (Mean ± SD or n [%])
Gender	Male	36 (63.2%)
	Female	21 (36.8%)
Age (years)	18~30	6 (10.5%)
	31~40	7 (12.3%)
	41~50	22 (38.6%)
	51~60	22 (38.6%)
Duration of hypertension	<1 year	8 (14.0%)
	1~5 years	23 (40.4%)
	6~10 years	12 (21.1%)
	>10 years	14 (24.6%)
Hypertension grade	Grade 1	15 (26.3%)
	Grade 2	24 (42.1%)
	Grade 3	18 (31.6%)
Antihypertensive medication intake	Regular	35 (61.4%)
	Intermittent	14 (24.6%)
	No medication	8 (14.0%)
Family history of hypertension	Yes	47 (82.5%)
	No	10 (17.5%)
Comorbidities	Diabetes	5 (8.8%)
	Hyperlipidemia	8 (14.0%)
	Hyperuricemia	1 (1.8%)
BMI (kg/m^2^)		26.99 ± 3.54
Current blood pressure (mmHg)	Systolic Blood Pressure	131.07 ± 13.15
	Diastolic Blood Pressure	88.61 ± 10.65

**Table 2:** Implicit association test instructions.

Trial Count	Task Type	Response for “E” Key	Response for “I” Key	Stimulus Ratio (Exercise:Positive:Negative)
24	Practice	Positive attribute OR Exercise	Negative attribute	7:7:10
72	Compatible task	Positive attribute OR Exercise	Negative attribute	7:7:10
24	Practice	Positive attribute	Negative attribute OR Exercise	7:10:7
72	Incompatible task	Positive attribute	Negative attribute OR Exercise	7:10:7

**Table 3:** Group differences in EEG entropy between exercisers and non-exercisers during affective IAT. z value (FDR-corrected *p* value).

Task Type	Brain Region	Entropy						
		Singular Spectrum	Approximate	Sample	Fuzzy	Permutation	Envelope	Log Energy
Compatible	Frontal	−1.541	−1.191	−1.141	−1.008	−0.475	−0.525	−0.358
		(0.818)	(0.818)	(0.818)	(0.818)	(0.818)	(0.818)	(0.818)
	Fronto-central	−1.274	−1.074	−0.908	−0.808	−0.558	−1.041	−0.858
		(0.818)	(0.818)	(0.818)	(0.818)	(0.818)	(0.818)	(0.818)
	Central	−0.941	−0.708	−0.525	−0.525	−0.308	−1.707	−1.157
		(0.818)	(0.818)	(0.818)	(0.818)	(0.818)	(0.818)	(0.818)
	Centro-parietal	−0.658	−0.208	−0.375	−0.558	−0.258	−1.341	−1.058
		(0.818)	(0.835)	(0.818)	(0.818)	(0.819)	(0.818)	(0.818)
	Parietal	−0.508	−0.341	−0.291	−0.641	−0.375	−0.541	−1.341
		(0.818)	(0.818)	(0.818)	(0.818)	(0.818)	(0.818)	(0.818)
Incompatible	Frontal	−1.990	−1.824	−2.057	−1.707	−1.207	−3.472	−0.758
		(0.274)	(0.284)	(0.274)	(0.308)	(0.345)	(0.012)	(0.507)
	Fronto-central	−1.840	−1.541	−1.790	−1.640	−1.224	−3.789	−0.858
		(0.284)	(0.328)	(0.284)	(0.321)	(0.345)	(0.000)	(0.467)
	Central	−1.357	−1.391	−1.474	−1.441	−0.874	−3.339	−0.841
		(0.332)	(0.332)	(0.328)	(0.328)	(0.467)	(0.012)	(0.467)
	Centro-parietal	−0.908	−1.041	−1.108	−1.307	−0.558	−2.390	−1.341
		(0.467)	(0.417)	(0.391)	(0.334)	(0.594)	(0.149)	(0.332)
	Parietal	−0.675	−0.691	−0.908	−1.274	−0.375	−1.474	−1.507
		(0.530)	(0.530)	(0.467)	(0.338)	(0.708)	(0.328)	(0.328)

**Table 4:** Group differences in EEG entropy between exercisers and non-exercisers during instrumental IAT. z value (FDR-corrected *p* value).

Task Type	Brain Region	Entropy						
		Singular Spectrum	Approximate	Sample	Fuzzy	Permutation	Envelope	Log Energy
Compatible	Frontal	−1.058	−0.608	−0.924	−1.357	−0.791	−0.824	−0.508
		(0.783)	(0.783)	(0.783)	(0.783)	(0.783)	(0.783)	(0.783)
	Fronto-central	−0.891	−0.708	−1.074	−1.324	−0.525	−0.425	−0.791
		(0.783)	(0.783)	(0.783)	(0.783)	(0.783)	(0.783)	(0.783)
	Central	−0.708	−0.525	−0.874	−1.174	−0.525	−0.241	−1.674
		(0.783)	(0.783)	(0.783)	(0.783)	(0.783)	(0.848)	(0.783)
	Centro-parietal	−0.441	−0.192	−0.458	−1.091	−0.291	−0.525	−1.557
		(0.783)	(0.848)	(0.783)	(0.783)	(0.848)	(0.783)	(0.783)
	Parietal	−0.192	−0.858	−0.275	−1.041	−0.425	−1.124	−1.307
		(0.848)	(0.783)	(0.848)	(0.783)	(0.783)	(0.783)	(0.783)
Incompatible	Frontal	−1.091	−0.741	−0.891	−1.324	−0.691	−0.425	−0.625
		(0.745)	(0.745)	(0.745)	(0.745)	(0.745)	(0.771)	(0.745)
	Fronto-central	−0.891	−1.174	−1.024	−1.357	−0.625	−0.491	−0.708
		(0.745)	(0.745)	(0.745)	(0.745)	(0.745)	(0.771)	(0.745)
	Central	−0.724	−0.924	−0.824	−1.241	−0.675	−0.941	−0.691
		(0.745)	(0.745)	(0.745)	(0.745)	(0.745)	(0.745)	(0.745)
	Centro-parietal	−0.408	−0.325	−0.675	−1.108	−0.458	−0.874	−0.475
		(0.771)	(0.780)	(0.745)	(0.745)	(0.771)	(0.745)	(0.771)
	Parietal	−0.325	−0.924	−0.591	−1.074	−0.308	−0.924	−0.208
		(0.780)	(0.745)	(0.746)	(0.745)	(0.780)	(0.745)	(0.835)

**Table 5:** Classification performance (%) across different machine learning models using envelope entropy.

Condition		Classifier Type	Accuracy		Sensitivity		Specificity		Precision		F1-Score	
			Mean	SD	Mean	SD	Mean	SD	Mean	SD	Mean	SD
Affective IAT	Compatible, incompatible	RF	70.0	6.5	53.9	12.6	81.6	10.8	69.6	17.3	59.2	11.3
		KNN	69.4	10.0	70.6	20.0	73.0	13.0	55.1	19.7	57.8	11.7
		SVM	62.5	11.4	63.3	17.7	68.5	21.9	51.9	26.5	50.5	11.4
		LDA	61.9	8.6	62.3	20.0	69.9	21.3	59.5	27.0	54.8	11.8
	Compatible	RF	56.9	10.8	60.3	28.9	59.7	11.5	29.0	18.9	37.8	15.6
		KNN	50.6	15.7	29.2	24.3	57.6	17.3	24.8	24.5	25.0	21.2
		SVM	50.0	5.9	11.7	12.9	56.5	10.5	8.7	16.4	24.4	7.7
		LDA	58.8	10.7	52.9	36.2	61.8	9.5	33.8	22.1	49.2	13.6
	Incompatible	RF	71.9	10.3	63.4	23.1	76.4	15.7	65.3	20.1	69.9	8.4
		KNN	74.4	8.6	66.4	17.7	81.8	12.2	73.6	18.5	67.1	11.5
		SVM	66.9	5.9	64.1	17.4	72.3	15.2	56.2	23.2	55.4	10.3
		LDA	61.9	12.7	73.4	21.7	60.2	16.1	41.1	16.9	49.5	12.4
Instrumental IAT	Compatible, incompatible	RF	50.0	10.6	32.3	16.8	59.4	11.5	27.0	16.5	31.3	13.2
		KNN	43.1	10.4	28.5	32.5	49.5	10.3	14.6	14.3	30.7	10.7
		SVM	48.8	8.2	13.3	11.5	51.8	10.7	4.2	9.0	20.2	2.9
		LDA	48.1	8.4	33.5	10.7	56.3	13.6	25.1	15.5	26.0	9.3
	Compatible	RF	50.0	16.1	33.1	23.9	56.0	17.0	32.4	22.8	40.5	17.3
		KNN	58.1	11.4	48.4	32.6	67.5	13.8	38.6	29.8	46.1	10.6
		SVM	45.0	8.7	16.7	19.2	54.9	11.1	16.4	23.9	25.2	8.9
		LDA	51.9	13.8	34.2	27.5	58.6	15.6	29.5	23.8	37.0	16.8
	Incompatible	RF	44.4	9.5	22.2	13.5	53.7	11.5	21.7	18.6	25.4	9.2
		KNN	50.6	8.6	42.0	29.3	55.3	10.1	23.1	19.8	32.7	11.0
		SVM	47.5	9.4	11.4	14.4	55.3	13.7	12.8	23.4	25.7	11.9
		LDA	48.1	10.2	19.4	22.7	56.4	11.7	15.4	21.5	31.5	16.8
Affective and instrumental IAT	Compatible, incompatible	RF	71.9	7.9	66.3	17.7	75.1	11.2	58.5	15.9	59.9	13.1
		KNN	68.8	6.6	64.6	17.6	72.0	8.8	53.1	13.5	56.3	9.9
		SVM	61.3	10.1	66.3	22.8	63.3	14.2	42.0	25.5	49.4	14.4
		LDA	59.4	12.2	57.9	27.1	61.5	14.4	42.2	21.7	45.6	18.8

## Data Availability

The data presented in this study are available from the corresponding author upon reasonable request due to privacy and ethical restrictions.

## Abbreviations

The following abbreviations are used in this manuscript:

IAT	Implicit association test
EEG	Electroencephalography
RF	Random forest
KNN	K-nearest neighbors
SVM	Support vector machine
LDA	Linear discriminant analysis

## Appendix A

**Table A1:** The included target words and attribute words (the mean for relevance and valence).

Word Type			Included Words					
Target			Exercise	Sport	Running	Jumping rope	Brisk walking	Cycling
		Relevance	8.38	7.81	7.75	7.44	7.38	7.38
Affective attribute	Positive		Pleasure	Vitality	Enjoyment	Satisfaction	Happiness	Joy
		Relevance	8.13	8.13	8.06	7.91	7.84	7.81
		Valence	8.06	7.97	7.47	7.63	7.94	7.91
	Negative		Disgust	Awful	Boring	Fatiguing	Painful	Uninteresting
		Relevance	8.00	7.72	7.63	7.59	7.59	7.56
		Valence	2.06	1.66	2.56	3.09	1.84	2.84
Instrumental attribute	Positive		Beneficial	Useful	Advantageous	Healthy	Important	Valuable
		Relevance	8.06	7.91	7.91	7.81	7.72	7.72
		Valence	7.81	7.44	7.66	7.91	7.31	7.97
	Negative		Ineffective	Useless	Harmful	Worthless	Meaningless	Unimportant
		Relevance	7.91	7.88	7.81	7.59	7.50	7.25
		Valence	2.22	2.25	1.81	1.88	1.88	3.34

## Appendix B

1. Singular spectrum entropy: This quantifies the complexity of EEG time series by analyzing the variance distribution of eigenvalues obtained from singular value decomposition.

$$SVDE_{n}=-\sum_{i=1}^{1}\frac{\lambda_{i}}{\sum \lambda }In\frac{\lambda_{i}}{\sum \lambda }$$

where $\lambda_{i}$ is the singular value of the diagonal matrix, and ∑λ are the traces of the diagonal matrix used to normalize the singular value.

2. Approximate entropy: It quantifies the regularity and predictability of EEG time series and is particularly suitable for signals with substantial random components.

$$AppxEn=\Theta^{m}-\Theta^{m+1}$$

$$\Theta^{m}=\frac{1}{N-m+1}\sum_{i=1}^{N-m+1}\log(\varepsilon_{i}^{m})$$

where the empirical dimension parameter m = 2 and the threshold ε = 0.15.

3. Sample entropy: This is an improved version of approximate entropy. It reduces estimation bias and minimizes the influence of signal length.

$$SampEn=-In\frac{P_{a}}{P_{b}}$$

where $P_{a}$ and $P_{b}$ are the conditional probabilities of occurrence of similar patterns in the sequences a and b of the signal, respectively.

4. Fuzzy entropy: This is a complexity measure derived from sample entropy theory. By introducing an exponential function to fuzzy the similarity measure, thereby overcoming the sensitivity of sample entropy to data length and discontinuous boundaries. Owing to the monotonicity and continuity of the fuzzy exponential function, fuzzy entropy varies smoothly and continuously with changes in parameters, allowing for the extraction of more detailed marginal information.

$$Fu\mathrm{En}(m,r)=\underset{x\to \infty }{\lim}[In\phi^{m}(r)-In\phi^{m+1}(r)]$$

where $\phi^{m}(r)=\frac{1}{N-m}\sum_{i=1}^{N-m}\left(\frac{1}{N-m-1}\sum_{j=1j\neq i}^{N-m}\mu (d_{ij}^{m+1},r)\right)$.

5. Permutation entropy: It evaluates the complexity of a time series by comparing neighboring values and mapping them into ordinal patterns. This approach effectively captures nonlinear dynamics, reduces the dimensionality of the problem space, and exhibits strong robustness to noise.

$$PermEn=-\sum_{i}^{D!}\rho_{\pi_{i}}In\rho_{\pi_{i}}$$

6. Envelope entropy: It measures signal complexity by quantifying the regularity of the amplitude envelope of a time series, thereby revealing dynamic characteristics related to temporal modulation of signal energy.

$$E_{p}=-\sum_{j=1}^{N}P_{j}\mathrm{lg}(P_{j})$$

$$P_{j}=a(j)/\sum_{j=1}^{N}a(j)$$

where *Pj* is the normalized form of *a*(*j*) and *a*(*j*) is the envelope signal obtained by Hilbert demodulation of the signal *y*(*j*).

7. Log energy entropy: It characterizes the complexity of EEG sub-bands, and quantify the variability of energy distribution across different frequency bands.

$$LogEn=\sum_{i=1}^{N}\log(y_{i}^{2})$$

where N is the length of the time series signal and $y_{i}$ is the sample of the signal.
